# Three Medicolegal Cases of Searching for the Stone: Lessons Learned Along the Journey

**DOI:** 10.5811/cpcem.2020.9.48652

**Published:** 2020-10-20

**Authors:** Ashley A. Jacobson, Ayumi E. Sakamoto, Gregory P. Moore, Eric T. Boie

**Affiliations:** * Mayo Clinic, Department of Emergency Medicine, Rochester, Minnesota; † Mayo Clinic, Kogod Center on Aging, Rochester, Minnesota

**Keywords:** Malpractice, kidney stone, contributory negligence

## Abstract

We present three medicolegal cases of medical negligence settled out of court. These cases all involved patients who presented to the emergency department (ED) with a suspected diagnosis of kidney stone. Highlighted are the importance of patient communication, addressing incidental findings found during a patient’s ED visit, anticipating complications, and the need for thorough documentation.

## INTRODUCTION

Many emergency physicians (EP) recognize the need for a complete laboratory evaluation and imaging to evaluate a patient’s presenting complaint within the emergency department (ED). Frequently, the importance of communicating with patients on incidental findings and ensuring they have a clear understanding of the discharge plan is not recognized. Complications of the diagnosis should also be anticipated and discussed. Three cases below illustrate how these factors can lead to medical negligence and financial liability. We also review the legal defenses of contributory negligence and comparative fault as a tool to decrease provider liability.

### CASE 1: *Kline versus St. Luke’s University Health Network et al*

A 49-year-old male with a history of kidney stones presented to the ED with severe back pain and trouble urinating. A computer axial tomography (CT) of the kidneys, ureter and bladder (KUB) was obtained and the study confirmed the presence of kidney stones in both kidneys, as well as a ureteral stone obstruction. In addition to the stones, the radiologist noted that there was a large hematoma or blood clot that could have been a mass in the bladder wall. The patient was discharged with a kidney stone diagnosis and advised to schedule an appointment with a urologist within three to five days. The patient returned a year later with worsening symptoms and was diagnosed with a tumor that had overtaken the majority of his bladder. Surgery to remove the tumor was unsuccessful.

Further review confirmed that it was the identical mass identified on CT a year earlier. The operation also revealed that the mass had evolved into an advanced cancer. The patient denied that he had been informed about the tumor when he was sent home with the diagnosis of kidney stone. Furthermore, he claimed that if he had been told about the mass, he would have more seriously heeded his doctor’s instructions to see a urologist. The patient brought suit as a plaintiff claiming that the delay and ambiguity of his diagnosis significantly decreased his chances of survival, in addition to subjecting him to another more invasive surgery. Although the defendants admitted that the radiologist’s notes indicated that the mass was potentially malignant, they contended that the patient was well informed about the mass and instructed to see a urologist, alleging that it was the patient’s very own negligence that caused the harm by not complying with the discharge orders. A settlement of 10 million dollars was rewarded.^1^

### CASE 2: *Anonymous versus Anonymous*

A 49-year-old female presented to the ED with abdominal pain. A CT was performed showing an obstructing kidney stone. A urinalysis was ordered by the EP to rule out urinary tract infection (UTI); however, nursing staff never obtained the specimen and the EP discharged the patient knowing that it had not been obtained. The patient presented a second time and ultimately died three days later from severe urosepsis secondary to an untreated UTI. A lawsuit was initiated for failure to complete the testing ordered, ultimately leading to delay in diagnosis and treatment, and subsequent death. The plaintiff argued that a urine sample is required for proper management of an obstructing kidney stone and that would have shown evidence of a UTI. The plaintiff claimed that if the diagnosis had been established on the first visit, she would have subsequently been treated with antibiotics, and death would have been prevented. The EP claimed a urine sample was attempted but was unsuccessful, and that even if the urinalysis had been completed, it might not have shown infection. A settlement of 2.6 million dollars was reached.^2^

### CASE 3: *Anonymous versus Anonymous*

A 35-year-old female presented to the ED with back pain. A CT KUB and urinalysis were completed demonstrating a kidney stone and concomitant UTI. Antibiotics and pain medication were prescribed. She was counseled to follow up with urology in two to four days. The patient went home under the impression she would pass the stone at home after discussion with the EP. Unfortunately, the pharmacy was closed and the patient did not fill the antibiotic medication that evening. The next morning, she was found confused and transported again to the ED in septic shock. The patient ultimately recovered after a two-week hospital stay that resulted in amputation of one forearm and bilateral feet. The plaintiff brought suit and argued that the importance of filling the prescription was not explained to her. The defense argued that she had received proper treatment and it was because of the patient’s negligence in not picking up her medication in a timely matter that she had a bad outcome. A settlement of 1.08 million dollars was reached.^3^

## DISCUSSION

### Ms. Sakamoto and Dr. Moore

These three cases illustrate some of the pitfalls and mistakes that can lead to increased liability for the EP. These may occur in the general ED diagnosis and treatment of patients of many diagnoses and specifically when evaluating for potential ureteral calculus.

Incidental CT findings are a common occurrence. A study by Thompson et al in 2011 reported that around 33.4% of 682 CTs performed in the ED of an urban Level I trauma center revealed at least one incidental finding. However, these findings were disclosed to the patient in discharge paperwork only 9.8% of the time. Alarmingly, some potentially life-threatening incidental findings such as aortic dilations and pulmonary nodules were only disclosed 33.3% and 25% of the time, respectively. In addition, patients were much less likely to receive disclosure if they did not have more than one incidental finding. Because this study only accounted for written disclosure on discharge paperwork, it is plausible that many patients were verbally informed of a finding. Nonetheless, the study reveals a lack of proper and thorough documentation and discharge instructions for patients with incidental findings.^4^

A similar study was conducted at another ED trauma center that considered the severities of incidental findings. Of 848 CTs, there were 289 incidental findings of varying severities. The incidental findings were classified as Category 1 (needing attention before discharge), Category 2 (requiring follow-up with a primary care provider in 1–2 weeks), or Category 3 (no follow-up needed). Of the 289 incidental findings, 31 were designated as Category 1, while 108 were Category 2, and 145 were Category 3. Only 48.4% of Category 1 incidental findings (15/31) had proper documentation of treatment, management, and follow-up.^5^ Collectively, these reports shed light on the significant lack of thorough disclosure and documentation of incidental CT findings in emergency care.

It is critical that ED patients are made aware of incidental findings and laboratory tests ordered from the ED. Every ED, hospital, radiology department, and laboratory should have defined mechanisms to identify and communicate abnormalities. The patient should be informed verbally of the importance of future studies, evaluation, and treatment. This should then be documented on the discharge instructions and in the chart, eg, “x-ray discrepancy discussed.” It is optimal if the primary physician or consultant can be informed as well.

When incidental findings or lab abnormalities are discovered after patient discharge, the information should be relayed to the patient via electronic, phone, or mailed communication, as well as to the primary physician or consultant, if possible, to avoid liability.

A safe “triangle” in these situations should be constructed between the following three points: 1) the EP; 2) the patient; and 3) the primary/consultant physician. When malpractice cases are pursued due to lack of communication of abnormal results that result in bad outcomes, the defendant is rarely successful in avoiding liability. The authors have identified multiple cases of abnormal tests that were not followed up and subsequently litigated. Not a single case reviewed was ruled in favor of the defendant.

### Dr. Jacobson and Dr. Boie

Nephrolithiasis typically presents as unilateral flank pain with radiation to the groin. The initial workup for first-time kidney stone typically consists of ordering a non-contrast CT abdomen/pelvis. A non-contrast CT abdomen/pelvis, at conventional radiation doses, is 94–97% sensitive and 96–99% specific for diagnosis of ureteral calculi and has become the gold standard for diagnosis when compared to renal and bladder ultrasound or abdominal radiography for first-time stone diagnosis.^6^ If there is concern for obstruction, a clean urine sample should be obtained for urinalysis and urine culture with concern for acute complicated UTI. If pyuria and bacteriuria are present, antibiotics should be administered and a urologic specialist consulted for consideration of possible surgical decompression.^6^,^7^

One single-center, prospective, observational study found that 7.8% of patients with kidney stones had concurrent UTI; this study did not comment on obstruction.^8^ Kidney stones account for approximately 66% of obstructive pyelonephritis.^7^ It is important to note that the urinalysis may appear deceptively normal if the infection is proximal to the obstruction.^7^,^9^ Additional risk factors that include perinephric fat stranding on CT, greater than 50 white blood cells per high-powered field on urine microscopy, a positive urine Gram stain, elevated procalcitonin, and elevated C-reactive protein can help risk stratify for possible proximal infection with a greater number of factors present increasing the likelihood of concurrent UTI.^9^ Obstructive kidney stone with subsequent pyelonephritis and sepsis has a 19% mortality rate without surgical decompression vs 9% mortality rate with intervention.^7^ As our cases illustrate, overwhelming urosepsis can occur rapidly, and admission to the hospital with prompt treatment may be optimal.

It is imperative to identify concomitant urine infection to decrease morbidity and mortality. In Case 3, the patient was prescribed antibiotics for a non-obstructed kidney stone with concurrent UTI, which is the standard of care. However, she was unable to pick up the medication from the pharmacy right away resulting in septic shock and significant morbidity of forearm and bilateral foot amputations.

Overall, discharged patients are prone to rapid deterioration from sepsis if concomitant infection is present. A first dose of antibiotics given in the ED, prior to discharge, would ensure initial compliance and theoretically improve patient outcomes. In general, urology consultation is recommended if a stone is obstructing the ureter and the urine is infected to insure optimal treatment and disposition for patient care.^3^

Contributory negligence is “conduct on the part of the plaintiff which falls below the standard to which he should conform for his own protection, and which is a legally contributing cause co-operating with the negligence of the defendant in bringing about the plaintiff’s harm.”^10^ In other words, if the patient is partly to blame for what happened then they may be assigned responsibility for the poor outcome as well as the care provider. This concept originated with the landmark legal case of *Butterfield v Forrester*.^11^ In this infamous case in 1809, a man was riding his horse extremely fast and was knocked off after hitting a pole that had recently been placed by the defendant. The jury determined that any person riding in a reasonable manner would have noticed the pole, thus avoiding the entire accident. Since the plaintiff’s own actions resulted in the accident, it was determined he should not be allowed to recover any damages.

Currently, four states (Alabama, Maryland, North Carolina, Virginia) and the District of Columbia recognize pure contributory negligence.^12^ If the patient is at all responsible for the negative outcome, they are not allowed to pursue their lawsuit. Most courts view contributory negligence as inherently unfair. These jurisdictions have replaced pure contributory negligence with comparative fault. This concept allows the plaintiff to recover some damages minus the plaintiff’s degree of fault. For example, a jury awards $100,000 to a case where the patient was deemed 90% at fault and the physician 10%. In this case, the plaintiff would only receive $10,000 (10%), which was the direct fault of the physician’s negligence.^12^

States have adopted different versions of comparative fault. Pure comparative fault as described above is recognized in 13 states. Eleven states recognize modified comparative fault – 50% bar, which allows the plaintiff to recover damages only if their percent at fault is less than or equal to 50%. If the plaintiff patient is more than 50% at fault, then he or she cannot recover any damages. Twenty-two states acknowledge modified comparative fault – 51% bar. In this rule, the plaintiff cannot recover damages if he or she is found to be greater than or equal to 51% at fault.^12^

For example, in *Harlow v Chin*, the patient was found 13% at fault for not seeking further medical workup for worsening pain from an undiagnosed cervical disc herniation ultimately leading to quadriplegia. This resulted in an award of 87% to the plaintiff.^13^ Looking at Case 1, the concept of comparative fault was advocated by the defense. At trial, the patient was determined to be 15% at fault, the EP 15%, and the emergency medicine resident physician 60%, thus awarding the patient $8.5 million from the $10 million verdict (ie, 85% determined due to be the direct negligence of the physicians’ malpractice).^1^ Overall, it is crucial for all incidental findings to be reported and clearly explained to the patient even if they had already been discharged from the ED.

Without proper systems in place to relay such findings, more patients will suffer and increased liability will ensue. Therefore, clear and effective communication is critical in the ED so that the patient is properly notified of any abnormal findings and advised to follow up appropriately. The physician should thoroughly inform the patient of the importance of a follow-up and the consequences of not doing so. Again, these disclosures should be well documented to ensure legal protection if patient outcomes are unfavorable.^12^ If this is accomplished, the defense of comparative fault or contributory negligence will be viable.

## CONCLUSION

We have presented three selected cases of claims of medical negligence that were settled before trial and not taken to court. They illustrate the importance of addressing incidental findings and communicating the results, and following up on laboratory ordering and result analysis. If proper communication and documentation are done by the provider, a legal defense of contributory negligence or comparative fault is a viable and accepted approach to avoiding successful litigation.

### Take-home Points

1. Incidental findings are not an infrequent occurrence when ordering CTs and subsequently become more health threatening to the patient than the primary medical issue. It behooves the physician to ensure follow-up of incidental findings.
2. It is extremely important to adequately document conversations, risks/benefits, and return precautions with the patient within written documentation.
3. It is vital to identify concomitant urine infection to decrease mortality in patients presenting with an obstructive kidney stone. Urology consultation is recommended if a stone is infected for surgical decompression and/or hospital admission. Consider first dose of antibiotics in the ED before discharge.
4. Be aware of the law in your state. Patients can be held responsible and assigned a percentage of fault if their action or inaction contributed to a poor outcome and harm.
  - Pure Contributory Negligence: Alabama, Maryland, North Carolina, Virginia, the District of Columbia
  - Pure Comparative Fault: Alaska, Arizona, California, Florida, Kentucky, Louisiana, Mississippi, Missouri, New Mexico, New York, Rhode Island, South Dakota, Washington
  - Modified Comparative Fault–50% bar: Arkansas, Colorado, Georgia, Idaho, Kansas, Maine, Nebraska, North Dakota, Tennessee, Utah, West Virginia
  - Modified Comparative Fault–51%bar: Connecticut, Delaware, Hawaii, Illinois, Indiana, Iowa, Massachusetts, Michigan, Minnesota, Montana, Nevada, New Hampshire, New Jersey, Oklahoma, Ohio, Oregon, Pennsylvania, South Carolina, Texas, Vermont, Wisconsin, Wyoming.
